# Generalisation of Placebo and Nocebo Effects: Current Knowledge and Future Directions

**DOI:** 10.1002/ejp.70018

**Published:** 2025-05-08

**Authors:** Lingling Weng, Kaya J. Peerdeman, Antoinette I. M. van Laarhoven, Andrea W. M. Evers

**Affiliations:** ^1^ School of Psychology Shenzhen University Shenzhen China; ^2^ Health, Medical and Neuropsychology Unit Institute of Psychology, Leiden University Leiden the Netherlands; ^3^ Leiden Institute for Brain and Cognition (LIBC) Leiden University Leiden the Netherlands; ^4^ Department of Psychiatry Leiden University Medical Center Leiden the Netherlands; ^5^ Medical Delta, Leiden University, Technical University Delft, Rotterdam University Rotterdam the Netherlands

**Keywords:** placebo and nocebo effects, response generalisation, somatic symptoms, stimulus generalisation, treatment outcomes

## Abstract

**Background and Objective:**

Placebo and nocebo effects are beneficial or adverse treatment outcomes upon administration of inert treatment components. These effects have been frequently studied on pain. It remains unclear to what extent generalisation occurs in these effects on pain and other somatic sensations. This review outlines the current knowledge on stimulus generalisation (i.e., generalisation over various stimuli) and response generalisation (i.e., generalisation over various responses) of placebo and nocebo effects on prevalent somatic sensations (i.e., pain, itch, dyspnea, nausea and fatigue).

**Databases and Data Treatment:**

The databases PubMed, Web of Science and PsycINFO were systematically searched for peer‐reviewed articles reporting on experimental studies in humans of the induction and generalisation of placebo and nocebo effects on prevalent somatic sensations.

**Results:**

Of 2025 records identified, 23 studies were included. These studies indicated that placebo and nocebo effects can generalise over stimuli (at perceptual, categorical and treatment levels) and over responses within modalities. Most studies investigated pain; fewer studies investigated itch, dyspnea, nausea and fatigue. Generalisation effects tend to be larger when the generalisation stimuli and responses more closely resemble the initial stimulus or response. Generalisation was more likely if a combination of verbal suggestion and conditioning was employed to induce placebo or nocebo effects than if either suggestion or conditioning was employed alone. Response generalisation across modalities remains unclear.

**Conclusions:**

Placebo and nocebo effects can generalise over stimuli and responses. More experimental and clinical research is warranted to address carryover effects of placebo and nocebo effects.

**Significance:**

The current review provides an overview of the literature on the generalisation of placebo and nocebo effects to diverse stimuli and responses. This can ultimately benefit healthcare providers to prevent carryover effects of treatment failure and harness carryover effects of treatment success.

## 1 Introduction

Carryover of previous treatment outcomes to subsequent medical treatments is pervasive. This learning feature is called generalisation, in which transferring prior experience to novel stimuli or situations is vital for survival in dynamic environments (Ghirlanda and Enquist 2003). Generalisation has been experimentally studied across diverse stimuli and responses, including emotion (e.g., fear) (Dymond et al. 2015), sensory perception (e.g., light, sound) (Ghirlanda and Enquist 2003) and behaviours (e.g., speech) (Ballard 2001). Recently, generalisation of treatment outcomes has started to attract attention in placebo and nocebo research (e.g., Carlino et al. 2016), reflecting the well‐known carryover effects of a patient's therapeutic history. Placebo and nocebo effects are beneficial or adverse effects, respectively, that occur in laboratory or clinical settings as part of active treatments or inert (placebo) treatment (Evers et al. 2018). These effects are driven by psychobiological mechanisms such as classical conditioning, affecting symptoms and treatment outcomes (Chavarria et al. 2017).

Studies of generalisation of placebo and nocebo effects can be categorised into stimulus generalisation and response generalisation. Generalisation at the level of stimulus occurs, for instance, when an individual who experiences pain relief from a placebo pill (conditioned stimulus) subsequently experiences the same pain relief (conditioned response) from other placebo treatments such as a placebo ointment (generalisation stimulus) (Zunhammer et al. 2017). This mimics a clinical scenario where experience with a treatment for one symptom may influence effects of other similar treatments for the same symptom. Generalisation at the level of response occurs, for instance, if an individual experiences pain alleviation (conditioned response) with a placebo device (conditioned stimulus), and subsequently also experiences alleviation of other symptoms, such as fatigue (generalisation response), with the same placebo device (Carlino et al. 2016). This mirrors a clinical situation where a positive treatment outcome on one symptom may predict or affect the outcome of that same treatment for other symptoms. It is crucial that generalisation occurs appropriately across stimuli and responses (Dunsmoor and Paz 2015). For instance, overgeneralisation of treatment failure may result in repeated failed treatment cycles.

Understanding generalisation in the placebo and nocebo field is essential to ensure that healthcare providers are aware of how patients' medical treatments history affects current and future treatment outcomes. However, a synthesis of the state of the art regarding generalisation of placebo and nocebo effects is lacking. This narrative review provides an overview of the literature on generalisation of placebo and nocebo effects on common somatic symptoms: pain, itch, dyspnea, nausea and fatigue (Wolters et al. 2019). We first review the current knowledge on the generalisation of placebo and nocebo effects and address behavioural and neuroimaging studies that demonstrate how these effects generalise. Following this, future recommendations are provided. Understanding these generalisation effects offers the possibility of amplifying carryover effects of therapeutic success and precluding carryover effects of therapeutics failure. Ultimately, this work could provide insight into treatment effects in complex clinical situations, such as for patients experiencing multiple symptoms simultaneously or those with multiple treatments that have had insufficient effects on chronic symptoms.

## 2 Method

After exploring the literature, the term placebo or nocebo effect was considered too restricted, as many studies investigated placebo‐ and nocebo‐like effects (Benedetti 2008). The difference between placebo/nocebo effects and placebo−/nocebo‐like effects is that the former follow‐ the administration of a placebo, whereas the latter occur without the administration of a placebo (Benedetti 2008). These effects affect symptoms and treatment outcomes, driven by similar mechanisms including expectancy learning. Therefore, studies that focused on the generalisation of placebo and nocebo as well as placebo‐ and nocebo‐like effects on somatic symptoms were considered for inclusion, i.e., the study procedure consisted of the induction of placebo and nocebo as well as placebo‐ and nocebo‐like effects and an examination of the generalisation of these effects. (Note that, in the remainder of this paper, we do not refer to placebo‐ and nocebo‐like effects unless of particular relevance). The common somatic symptoms are pain, itch, dyspnea, nausea and fatigue (these symptoms were selected building on a previous review (Wolters et al. 2019)). Generalisation of placebo and nocebo effects was operationalised as a phenomenon in which placebo and nocebo effects that are experimentally induced with one stimulus/response are then seen to transfer to another stimulus/response (Shepard 1957). Publications were included if they were primary peer‐reviewed journal articles describing experimental studies conducted in humans. The included studies reported valid measures of somatic symptoms.

Systematic searches in PubMed, Web of Science and PsycINFO were conducted until 15 September 2022 (no time limitation by year). The searches were rerun on 20 January 2024. To broaden the search scope, the search strategy for placebo (/−like) or nocebo (/−like) effects used all terms related to conditioning and verbal suggestion that are the most common learning procedures used in placebo/nocebo research, since studies that employed conditioning or verbal suggestion did not always use the terms placebo (/−like) or nocebo (/−like) effects. Therefore, the following (and related) search terms were used: placebo and nocebo effects (conditioning, observe* learning, verbal suggestion, learning, expecta*), in combination with search terms for generalisation (generali*, transfer*, carryover) and somatic symptoms (pain, analgesi*, hyperalgesia*, hypoalgesia, fatigue*, itch*, prurit*, antipruritic*, nause*, motion sick*, emetic*, antiemetic, dizziness*, vertigo, syncope, faint*, dyspnea*, asthma*) (the full keyword profile per database is reported in Appendix). One reviewer (L.W.) screened titles and abstracts of all records identified and subsequently assessed the full text of the papers selected for eligibility. For each eligible study, data were extracted regarding authors, year of publication, intervention, study design, main outcome and measures. The information extracted was filled out by one author (L.W.) and checked by one of the other authors (K.J.P. or A.I.M.v.L.). When sufficient data were available, effect sizes, standard errors (SE) and 95% confidence intervals (CI) were calculated. These analyses were conducted by the first reviewer (L.W.) and checked by two reviewers (K.J.P. and LB), using Comprehensive Meta‐Analysis software, version 4.0.000 (Biostat, Englewood, CO). Risk of bias (RoB) for each study included was assessed by a research assistant and one of the authors (L.W.) using the revised Cochrane risk‐of‐bias tool (version 2, 22 August 2019). The assessment covered five domains: ‘bias arising from the randomization process’, ‘bias due to deviations from intended interventions’, ‘bias due to missing outcome data’, ‘bias in measurement of the outcome’, ‘bias in selection of the reported result’. Additionally, for crossover studies, an extra domain—‘bias arising from period and carryover effects’—was evaluated. Any disagreements were resolved by a third author (A.W.M.E.). The review narratively analysed the results and deduced the common findings from the studies included. The studies included were too few in number and too heterogeneous in nature to warrant a meta‐analysis.

## 3 Results

**FIGURE 1:**
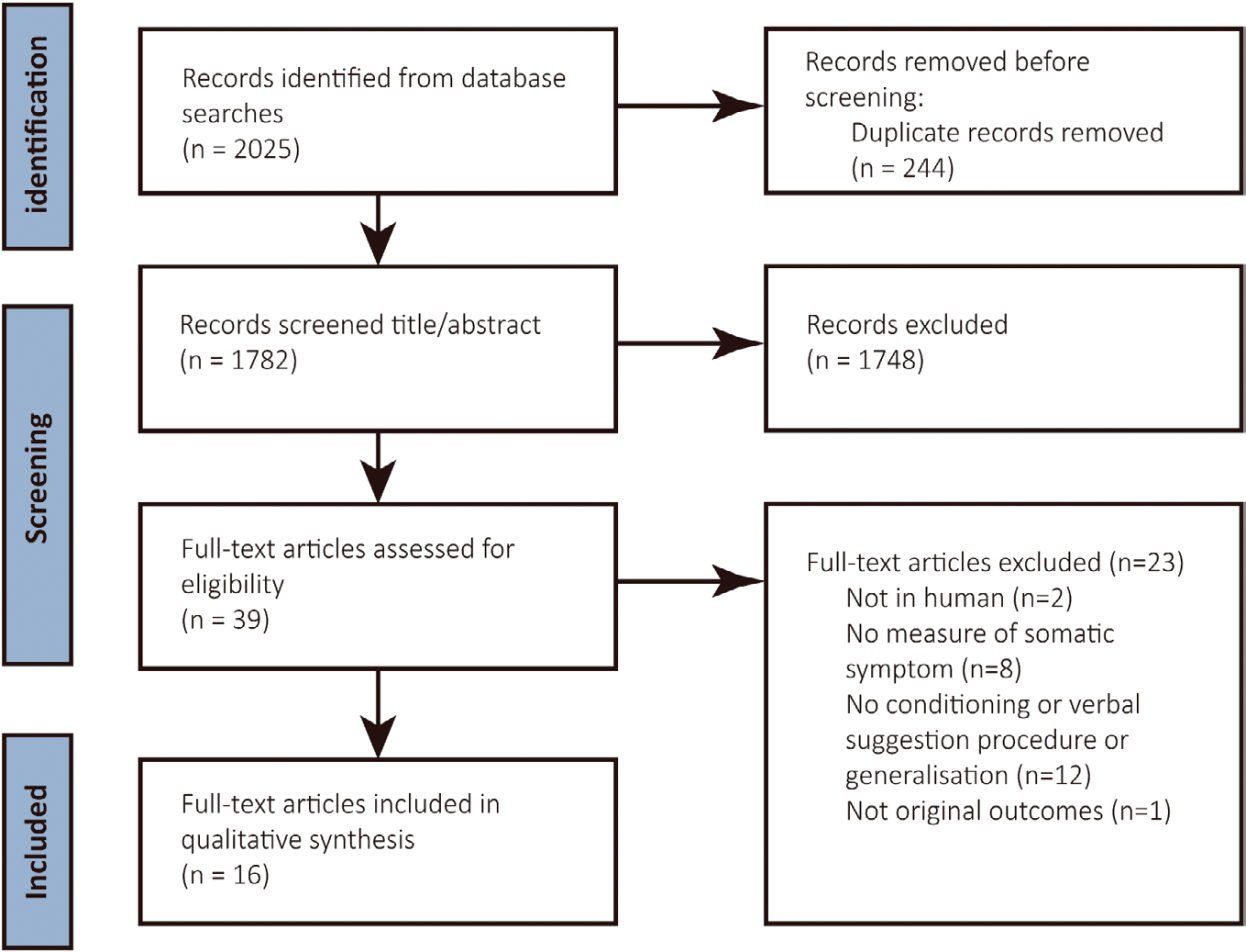
Flow diagram of literature search.

As Figure 1 shows, 1913 records were identified in the three databases. After the removal of duplicates and the subsequent screening of titles and abstracts, 34 records were included for full‐text evaluation. When rerunning the searches in the databases in January 2024, 112 new records were inspected, during which 5 records were included for full‐text evaluation. In total, of these 39 records, 2 records were excluded because the experiments investigated animals (Iguchi et al. 2014; Molecolare et al. 1986), 8 records were excluded due to not measuring somatic sensations (e.g., only expectancy of somatic sensations was rated) (Biggs et al. 2020; Janssens et al. 2015, 2017, 2020; Kloos et al. 2022; Trost et al. 2008; Tu et al. 2021; Zhao et al. 2015), 12 records were excluded due to the absence of a procedure related to either the induction of placebo‐ or nocebo(−like) effects or generalisation (Bush et al. 2023; Garrick‐Bethell et al. 2008; Glogan et al. 2023; Godfrey et al. 2017; Hanssen et al. 2014; Huang et al. 2023; Morton et al. 2010; Shafir et al. 2023; Sharpe et al. 2015; Vandael et al. 2023; Veldman et al. 2015; Zunhammer 2018), and 1 record was excluded because the results presented overlapped with part of another record included (Kessner et al. 2013). In total, 16 articles were included, reporting on 23 studies. The overview of the studies included is provided in Table 1 (generalisation over stimuli) and in Table 2 (generalisation over responses).

**TABLE 1:** Overview of the studies included testing stimulus generalisation of placebo and nocebo (and placebo‐ and nocebo‐like) effects on prevalent physical symptoms.

Study	N*	Intervention	Conditioned stimulus	Control stimulus	Unconditioned stimulus	Learning mechanism; conditioning paradigm if applied; total number of trials	Generalisation stimulus (at the perceptual/categorical/treatment level); total number of trials	Timing of generalisation test	Original and generalisation response and measure
Choi et al. (2022)	30; B	Placebo	A placebo cream	A control cream	Heat	Verbal suggestion + conditioning; placebo group: placebo cream +40°C; control cream +44.5°C; control group: placebo cream +43.5°C; control cream +44.5°C; 20	# (treatment level) cold‐pack +44.5°C; 10.	Immediate	Pain; 0–100 pain NRS
Devriese et al. (2000)	56; W + B	Placebo/nocebo‐like	Ammonia/niaouli; one as conditioned stimulus CS+, and the other as control stimulus CS−		CO_2_ inhalation	Conditioning; CS+ paired with CO_2_‐enriched air; CS− paired with regular air; 20	(perceptual level) new odours (butyric acid, acetic acid, & citric aroma) + regular air; 3	Immediate & a week delay	Dyspnea; 16‐item symptom checklist
Hofmann et al. (2014)	30; W + B	Placebo	An inert patch (treated site, CS_treated_)	No treatment (untreated site, CS_untreated_)	Heat	Verbal suggestion + conditioning; placebo group: CS_treated_ + low pain; CS_untreated_ + high pain; 40. Control group: CS_treated_ + high pain; CS_untreated_ + high pain; 40.	# (treatment level) inert oral pill, treated site + medium pain; untreated site + high pain; 30	Consecutive days (days 1 & 2 conditioning, day 3 test)	Pain; 0–100 pain VAS
Kampermann et al. (2021) experiment 1	18; W	Placebo	A facial image (CS+)	The most dissimilar facial image (CS−)	Heat	Verbal suggestion + conditioning; CS+ face + low pain; CS− face + moderate pain; 20	(perceptual level) 6 facial images with novel angles + moderate pain;36	Immediate	Pain; 0–100 pain VAS
Kampermann et al. (2021) experiment 2	39; W	Placebo	A facial image (CS+)	The most dissimilar facial image (CS−)	Heat	Verbal suggestion + conditioning; CS+ face + low pain; CS− face + moderate pain; 20	(perceptual level) 6 facial images with novel angles + moderate pain;36	Immediate (day 1 calibration, day 2 calibration + conditioning + test in MRI scanner)	Pain; 0–100 pain VAS
Kessner et al. (2014)	39; W + B	Placebo	An inert patch (treated site, CS_treated_)	No treatment (untreated site, CS_untreated_)	Heat	Verbal suggestion + conditioning; Placebo group: CS_treated_ + low pain; CS_untreated_ + high pain; 40. Control group: CS_treated_ + high pain; CS_untreated_ + high pain; 40.	# (treatment level) inert ointment at treated site + medium pain; untreated site + high pain; 30	Consecutive days (days 1 & 2 conditioning, day 3 test in MRI scanner)	Pain; 0–100 pain VAS
Koban et al. (2018) experiment 1	38; W	Placebo‐and nocebo‐like	Two Gabor patches with different orientation angles (one as CS_low_ and the other as CS_high_)		Heat	Conditioning; Placebo‐like group: CS_low_ + low or medium pain; Nocebo‐like group: CS_high_ + high or medium pain; 80	(perceptual level) 11 novel Gabor angles + medium pain; 55	Immediate	Pain; 0–100 pain VAS
Koban et al. (2018) experiment 2	60; W	Placebo‐and nocebo‐like	Two Gabor patches with different orientation angles (one as CS_low_ and the other as CS_high_)		Heat	Conditioning; Placebo‐like group: CS_low_ + low or medium pain; Nocebo‐like group: CS_high_ + high or medium pain; 96	(perceptual level) 11 novel Gabor angles + medium pain; 44	Immediate	Pain; 0–100 pain VAS
Koban et al. (2018) experiment 3	36; W	Placebo‐and nocebo‐like	An animal drawing & a vehicle drawing; one as conditioned stimulus CS+, and the other as control stimulus CS−		Heat	Conditioning; Placebo‐like group: CS_low_ + low or medium pain; Nocebo‐like group: CS_high_ + high or medium pain; 80	(categorical level) 18 novel animal & vehicle (by visually presenting in drawings, photos and words) + medium pain; 90	Immediate	Pain; 0–100 pain VAS
^a^ Liu et al. (2019) experiment 1	56; W	Placebo‐and nocebo‐like	8 animal & 8 tool images (CS_low_ & CS_high_)	16 fruit images (CS_control_)	Electrocutaneous	Conditioning; CS_low_ + low pain; CS_high_ + high pain;32	(categorical level) 16 novel animal & tool images + medium pain; 16	Immediate	Pain; 1–9 pain NRS
^a^ Liu et al. (2019) experiment 2	42; W	Placebo‐ and nocebo‐like	An animal & a tool image (CS_low_ & CS_high_)	9 fruit images (CS_control_)	Electrocutaneous	Conditioning; CS_low_ + low pain; CS_high_ + high pain;32	(categorical level) 16 novel animal & tool images + medium pain; 16	Immediate	Pain; 1–9 pain NRS
Quinn et al. (2015)	30; B	Nocebo	A context (i.e., a testing room)	n/a	Active galvanic vestibular stimulation (inducing nausea)	Conditioning: Control group: a context + control stimuli; Nocebo group: a context + active stimuli; Nocebo‐context‐change group: a novel context + active stimuli; 25 min for each stimulus	(treatment level) the context used in nocebo group in conditioning + control stimuli; 25 min for each stimulus	Non‐consecutive days (days 1 & 2 conditioning, day 3 test)	Nausea; 0–10 nausea‐related symptoms VAS
^b^ Zunhammer et al. (2017)	186; W + B	Placebo	An inert cotton patch (treated site, CS_treated_)	No treatment (untreated site, CS_untreated_)	Heat	Verbal suggestion + conditioning; Placebo group: CS_treated_ + low pain; CS_untreated_ + high pain; 40. Control group: CS_treated_ + high pain; CS_untreated_ + high pain; 40.	# (treatment level) inert ointment/novel patch at treated site + medium pain; untreated site + high pain; 30; oral pill/tablet, treated site + medium pain; untreated site + high pain; 30	Consecutive days (days 1 and 2 conditioning, day 3 test)	Pain; 0–100 pain VAS

*Note:* All studies included samples of healthy participants only.

Abbreviations: #, using verbal suggestion on generalisation stimuli; *B, between subjects design; W, within subjects design; W + B, within‐between subjects design; CS, conditioned stimulus; MRI, magnetic resonance imaging; NRS, numeric rating scale; VAS, Visual Analog Scale.

^a^ In Liu's study, three categories (i.e., animal, tool and fruit) paired with low, high and medium pain intensities were counterbalanced across participants for CS_low_, CS_high_ and CS_control_. The table shows an example of CS_low_ and CS_high_ as well as CS_control_.

^b^ Zunhammer's study combined and analysed the data from six substudies, including the studies by Kessner and Hofmann, as well as other, unpublished studies. Note that the findings were reported per group for the studies including both placebo and nocebo groups.

**TABLE 2:** Overview of the studies included testing response generalisation of placebo and nocebo effects on prevalent physical symptoms.

Study	N*	Intervention	Conditioned stimulus	Control stimulus	Unconditioned stimulus	Learning mechanism; conditioning paradigm if applied; total number of trials	Generalisation response (within/across modality); total number of trials	Timing of generalisation test	Original response and measure	Generalisation response and measure
^a^ Bartels et al. (2017)	99; W	Nocebo and subsequent placebo‐counterconditioning	A sham electrode ON	A sham electrode OFF	Electrocutaneous	Verbal suggestion + conditioning; part 1 nocebo group: ON + high electrical itch; OFF + medium electrical itch; part 2: placebo group, ON + low electrical itch; OFF + medium electrical itch; nocebo group, ON + high electrical itch; OFF + medium electrical itch; control group, ON + medium electrical itch, OFF + medium electrical itch; 16 trials per group and part.	# (within modality) Histamine‐evoked itch+ ON;1 for each group	Immediate	Itch (electrical itch); 0–10 itch NRS	Itch (histamine‐induced itch); 0–10 itch NRS
Carlino et al. (2016) experiment 1	40; B	Placebo	Two sham electrodes ON	No electrode	Electrocutaneous	Verbal suggestion + conditioning; ON + low pain	# (across modalities) motor endurance + electrodes ON, motor endurance without electrodes; Note that the number of motor trial repetitions was the DV.	Consecutive days (day 1 calibration, days 2 & 3 conditioning, day 4 generalisation test)	Pain (electrical pain); The duration of the test reaches 10 on 0–10 pain NRS or participants ask to stop	Fatigue (motor repetitions); The number of move repetitions until complete exhaustion by using 0–10 fatigue NRS
Carlino et al. (2016) experiment 2	40; B	Placebo	Two sham electrodes ON	No electrode	Electrocutaneous	Verbal suggestion	# (across modalities) motor endurance + electrodes ON, motor endurance without electrodes; Note that the number of motor trials was the DV.	Different days (day 1 calibration, day 4 generalisation test)	Pain (electrical pain); The duration of the test reaches 10 on 0–10 pain NRS or participants ask to stop	Fatigue (motor repetitions); The number of move repetitions until complete exhaustion by using 0–10 fatigue NRS
Voudouris et al. (1989)	20; W	Placebo and nocebo	A placebo cream	No cream	Iontophoretic pain generator	Verbal suggestion + conditioning; placebo group: placebo cream + low pain; no cream + medium pain,6; nocebo group: placebo cream + high pain; no cream + medium pain; 6	# (within modality) placebo cream + ischemic pain; no cream + ischemic pain; 2 for each group	Consecutive days	Pain (iontophoretic pain); 5 points pain analogue records (change in iontophoretic pain pre‐ versus post‐conditioning)	Pain (ischemic pain); 5 points pain analogue records (change in ischemic pain pre‐ versus post‐conditioning).
^b^ Weng et al. (2022b)	65; W	Placebo and nocebo	A sham TENS device ON	A sham TENS OFF	Heat	Verbal suggestion + conditioning; placebo group: ON + low heat pain; OFF + medium heat pain; 30; nocebo group: ON + high heat pain; OFF + medium heat pain; 30	(within modality) medium pressure pain + ON; medium pressure pain + OFF; 6. (across modalities) medium cowhage‐evoked itch + ON; medium cowhage‐evoked itch + OFF; 2	Immediate	Pain (heat pain); 0–10 pain NRS	Pain (pressure pain) and itch (cowhage‐evoked itch); 0–10 pain NRS (for 1st generalisation response); 0–10 itch NRS (for 2nd generalisation response)
^b^ Weng et al. (2022a)	44; W	Nocebo	Placebo solution	Control solution	Cowhage spicules	Verbal suggestion; nocebo solution + low cowhage‐evoked itch; control solution + low cowhage‐evoked itch; 2	(within modality) low mechanical itch + nocebo solution; low mechanical itch + control solution; 6. (across modalities) Mechanical stimuli intended to evoke touch at baseline + nocebo solution; mechanical touch + control solution; 6.	Immediate	Itch (cowhage‐evoked itch); 0–10 itch VAS	Itch (mechanical itch) and touch (mechanical touch); 0–10 itch NRS (for both generalisation responses)
^c^ Zhang and Luo (2009) experiment 1	16; W	Placebo	A sham electrode ON	A sham electrode OFF	CO_2_ laser stimulator	Verbal suggestion+ conditioning; ON + low laser‐evoked pain; OFF + high laser‐evoked pain; 24	# (across modalities) Unpleasant images + ON; Unpleasant images + OFF; 36	Immediate	Pain (laser pain); 0–100 pain VAS	Negative emotion (picture‐induced unpleasantness); 0–100 unpleasantness scale
Zhang and Luo (2009) experiment 2	20; W	Placebo	A sham electrode ON	A sham electrode OFF	CO_2_ laser stimulator	Verbal suggestion + conditioning; ON + low pain; OFF + high pain; 24	# (across modalities) Unpleasant images + ON; Unpleasant images + OFF; 200.	Immediate	Pain (laser pain); 0–100 pain VAS	Negative emotion (picture‐induced unpleasantness); 0–100 unpleasantness scale
Zhang and Luo (2009) experiment 3	24; W	Placebo	A sham electrode ON	A sham electrode OFF	CO_2_ laser stimulator	Verbal suggestion + conditioning; ON + low pain; OFF + high pain; 24	# (across modalities) Unpleasant images + ON; Unpleasant images + OFF; 12 for half of participants and 20 for the other half.	Immediate	Pain (laser pain); 0–100 pain VAS	Negative emotion (picture‐induced unpleasantness); 0–100 unpleasantness scale
^d^ Zhang et al. (2011)	27; W	Placebo	A sham electrode ON	A sham electrode OFF	CO_2_ laser stimulator	Verbal suggestion + conditioning; ON + low pain; OFF + high pain; 24	# (across modalities) Unpleasant images+ ON; unpleasant images+ OFF; 8.	Immediate	Pain (laser pain); 0–10 pain scale	Negative emotion (picture‐induced unpleasantness); 0–10 unpleasantness scale

*Note:* All studies included samples of healthy participants only. ON refers to the supposed activation of the sham electrode/device and OFF refers to the supposed deactivation of the sham electrode/device, although no electrodes/devices were active in the experiment.

Abbreviations: #, using verbal suggestion on generalisation responses; *B, between‐subject design; W, within‐subject design; DV, dependent variable; NRS, Numeric Rating Scale; VAS, Visual Analogue Scale.

^a^ Note that the design consisted of part 1 and part 2.

^b^ Note that the studies investigated response generalisation both within modality and across modalities, and the results of response generalisation within and across modalities were presented separately.

^c^ Note that in Zhang's paper (2009), one group in experiment 1 was not shown because no conditioning or verbal suggestions were given on initial laser pain, and only verbal suggestions were used on the generalisation response ‘emotion’; this does not meet our inclusion criteria.

^d^ Note that we deduced the results of the generalisation effects in a behavioural experiment from figures, as the behavioural experiment was only used for selecting the placebo responders (who learned placebo effects) to join the subsequent fMRI study and did not report initial placebo effects and generalisation effects. The findings of the generalisation effects in the fMRI study were not reported because the sample was limited to placebo responders (who learned placebo effects). Note that the findings were reported per group if the studies included both placebo and nocebo groups.

### 3.1 Risk of Bias

**FIGURE 2:**
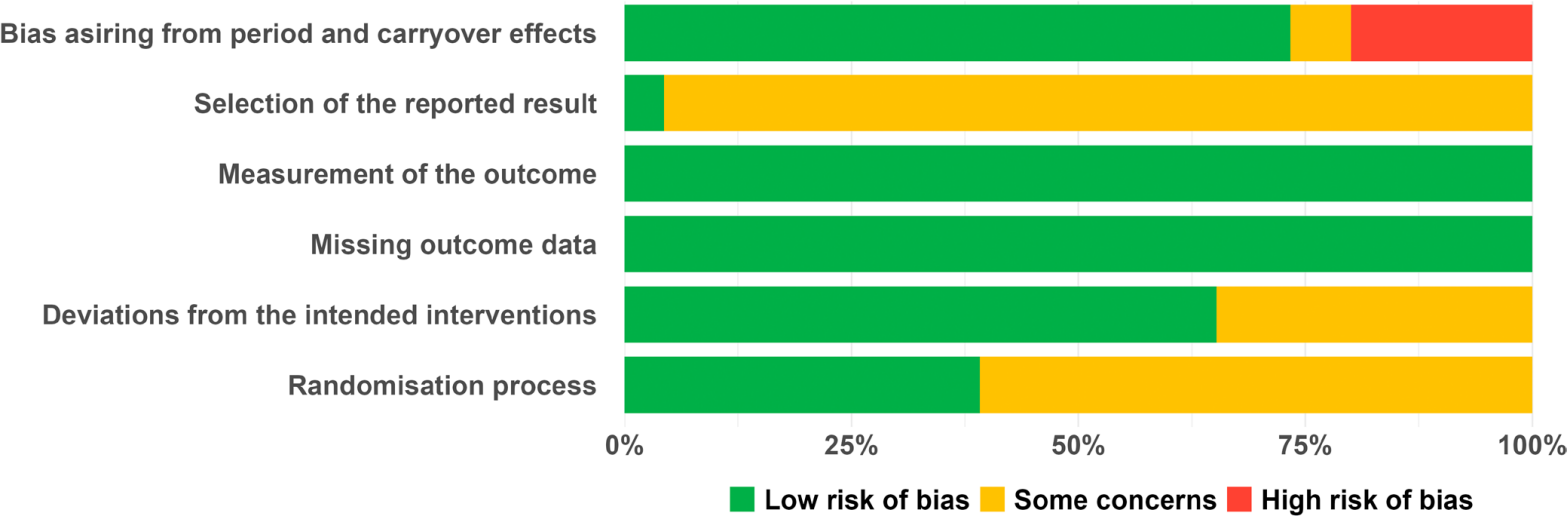
Risk of bias summary: Judgements on each risk of bias domain presented as percentages per study, covering both stimulus and response generalisation of placebo and nocebo effects.

The results of the RoB assessment for all included studies per domain are presented in Figure 2, and individual study assessment for both types of generalisation studies is presented in Figures S1 and S2. Across the included studies, most domains were rated as having a low risk of bias, though a judgement of some concerns was common regarding bias in the selection of the reported results (22/23) and the randomization process (14/23), indicating potential methodological limitations. Studies with some concerns regarding the selection of the reported result did not specify whether analyses followed a pre‐specified plan; additionally, 5 of these studies also did not explicitly address whether the results were based on data from both periods or whether carryover effects were identified. Regarding the randomization process, studies about which there were some concerns did not report an adequate concealment procedure. Studies about which there were some concerns regarding bias due to deviation from intended interventions did not clarify whether deviations arose due to the trial context. A judgement of a high risk of bias arising from period and carryover effects was made for 3 studies, which was primarily attributed to insufficient time to fully eliminate carryover effects related to emotion evoked by images. When analysed by generalisation type, the randomization process was predominantly rated as giving rise to some concerns for stimulus generalisation experiments (11/13) but most frequently as having a low of bias for response generalisation experiments (7/10). Conversely, some concerns about deviations from intended interventions were more common in response generalisation studies (6/10) than in stimulus generalisation studies (2/13).

### 3.2 Procedure to Test Generalisation of Placebo and Nocebo Effects and Types of Generalisation

The basic design of studies testing generalisation of placebo and nocebo effects consists of two parts: induction (usually with a testing phase for these effects) and generalisation. Specifically, induction generally uses expectancy‐induction methods, including verbal suggestion and classical conditioning (Blythe et al. 2019; Colagiuri et al. 2015). Verbal suggestion can induce and modify participants' expectations regarding outcomes by explicitly providing them with information (Blythe et al. 2019; De Pascalis et al. 2002). For instance, participants were told that a placebo cream, with supposedly an active component, could relieve pain (Weng et al. 2022b). Classical conditioning is a form of associative learning. An association is repeatedly made between a placebo or nocebo stimulus and a stimulus that evokes symptoms (unconditioned stimulus, inducing an unconditioned response). This may result in a change in perceived symptom intensity (conditioned response) when the placebo or nocebo stimulus is presented alone (conditioned stimulus) (Rescorla 1988; Rescorla 1996). After induction (and testing) of placebo and nocebo effects, two types of generalisation are tested: (1) stimulus generalisation and (2) response generalisation. To assess generalisation of the level of stimulus, the extent is measured to which novel, generalisation stimuli (e.g., a placebo pill) evoke responses (e.g., pain relief) similar to responses to conditioned stimuli (e.g., an ointment) (Andreatta et al. 2015; Shepard 1957, 1958; Shepard and Chang 1963; Till and Priluck 2000). To test generalisation at the level of response, the extent is measured to which novel, generalisation responses (e.g., reduced headache) akin to the original conditioned response (e.g., reduced muscle pain) occur with the same conditioned stimulus (e.g., an ointment) (Shepard 1958; Till and Priluck 2000; Weng et al. 2022b). The design of studies testing generalisation of placebo‐ and nocebo‐like effects generally consists of conditioning and generalisation, without placebo treatments and verbal suggestions (on placebo treatments) being provided. When testing each type of generalisation, the effects on behavioural and/or neurobiological response are assessed. In some studies, predictors for generalisation effects are also investigated. All studies included samples of healthy participants only. Self‐reported sensations ratings were the main outcome of interest in all studies.

#### 3.2.1 Stimulus Generalisation of Placebo (−Like) and Nocebo (−Like) Effects

Of 13 studies investigating generalisation of placebo and nocebo (and placebo‐ and nocebo‐like) effects to similar but novel stimuli (Table 1), 11 studies first induced placebo and/or nocebo (and placebo‐ and/or nocebo‐like) effects on pain, evoked in nine studies by heat stimuli and in two studies by electrical stimuli; one study assessed the effects on dyspnea; one study on nausea. Among the 13 studies, six studies focused on stimulus generalisation of placebo effects, one study on generalisation of nocebo effects, and the remaining six studies on generalisation of placebo‐ and/or nocebo‐like effects. Of these 13 studies, five studies investigated stimulus generalisation at the treatment level (from an initial placebo/nocebo treatment or treatment context to novel forms of placebo/nocebo treatment or treatment context), five studies investigated stimulus generalisation at the perceptual level (from initial Gabor patch/facial image/odour to novel Gabor patches/facial images/odours), and three studies investigated stimulus generalisation at the categorical level (from initial images of animal/tool/vehicle to novel images in the same categories). Effect sizes for each study included are presented in Table 3.

**TABLE 3:** Overview of the findings of the studies included testing stimulus generalization of placebo and nocebo (and placebo‐ and nocebo‐like) effects on prevalent physical symptoms.

Study	Findings of original induced effects	Findings of generalization effects	Std difference in means	SE	95%CI	Overall risk of bias
Choi et al., (2022)	Initial placebo effects were observed.	No generalization of placebo effects on pain from a placebo cream to a cold‐pack was observed (i.e., pain ratings were significantly higher with the cold‐pack in the placebo group than in the control group). Note that the findings of fMRI measures are discussed on the section 3.1.1.	−0.827	0.38	[−1.585, −0.068]	Some concerns
Devriese et al., (2000)	Conditioning effects were only observed when ammonia worked as conditioned stimulus.	Generalization of conditioning effects on dyspnea was only observed in butyric acid and acetic acid (foul‐smelling), only when participant acquired conditioning effects and in participants who reported high negative affect (i.e., total symptoms were higher in butyric acid and acetic acid, but not in citric aroma).	NA	NA	NA	Some concerns*
Hofmann et al., (2014)	Initial placebo effects were not reported.	No generalization of placebo effects on pain from an inert patch to an inert oral pill was observed (i.e., pain intensities were not significantly lower with the inert patch in the placebo group than in the control group).	0.04	0.365	[−0.676, 0.756]	Some concerns
Kampermann et al., (2021) experiment 1	Initial placebo effects were observed.	Generalization of placebo effects on pain across a facial image various angles was observed (placebo pain relief was strongest for CS+ and decreased with decreasing similarity to CS‐)	NA	NA	NA	Some concerns*
Kampermann et al., (2021) experiment 2	Initial placebo effects were observed.	Generalization of placebo effects on pain across a facial image various angles with various angles was observed (placebo pain relief was strongest for CS+ and decreased with decreasing similarity to CS‐.) (note that MRI results were not reported in the publication).	NA	NA	NA	Some concerns*
Kessner et al., (2014)	Initial placebo effects were not reported.	Generalization of placebo effects on pain from an inert patch to an inert ointment was observed (i.e., pain intensity was lower with the inert ointment in the placebo group than in the control group). (Note that fMRI results are discussed on the section 3.1.1.)	3.383	0.499	[2.404, 4.361]	Some concerns
Koban et al., (2018) experiment 1	Initial conditioning effects were observed	No generalization of conditioning effects on pain across Gabor angles was observed, but generalization effects were observed for the subgroup of participants who learned conditioning effects.	0.188	0.164	[−0.133, 0.509]	Some concerns*
Koban et al., (2018) experiment 2	Initial conditioning effects were observed	Generalization of conditioning effects on pain across Gabor angles was observed. (Pain ratings were increased as increasing similarity to CShigh. Pain ratings were increased as increasing dissimilarity to CSlow on one side of lager angles)	0.293	0.132	[0.035, 0.551]	Some concerns*
Koban et al., (2018) experiment 3	Initial conditioning effects were observed	Generalization of conditioning effects on pain was observed in the same conceptual category. (pain ratings were higher in CShigh category than in CSlow category). (stronger generalization effects for the CS learned concept (e.g.,’dog’ as CSlow) than novel concept (e.g., ‘cow’)).	0.36	0.172	[0.023, 0.697]	Some concerns*
^a^ Liu et al., (2019) experiment 1	Initial conditioning effects were observed.	Generalization of conditioning effects on pain was observed in the same conceptual category. (i.e., no difference in pain ratings between the conditioned stimuli and the generalization stimuli).	Placebo effects			Some concerns*
			0.089	0.134	[−0.173, 0.352]	
			Nocebo effects			
			‐0.134	0.134	[−0.397, 0.129]	
^a^ Liu et al., (2019) experiment 2	Initial conditioning effects were observed.	Generalization of placebo‐like effects on pain was observed in the same conceptual category. (i.e., no difference in pain ratings between the conditioned stimuli and the generalization stimuli), while no generalization of nocebo‐like effects was observed (i.e., a significant difference on pain ratings between the conditioned stimuli and the generalization stimuli).	Placebo effects			Some concerns*
			0.116	0.155	[−0.188, 0.419]	
			Nocebo effects			
			‐0.232	0.156	[−0.538, 0.075]	
Quinn et al., (2015)	Initial nocebo effects were observed.	Generalization of nocebo effects on nausea in one context to a novel context was observed (i.e., nausea ratings did not differ between the two contexts.)	NA	NA	NA	Some concerns
^b^ Zunhammer et al., (2017)	Initial placebo effects were not reported.	Generalization of placebo effects on pain from an inert patch to an inert ointment/novel patch was observed (i.e., pain intensities were lower with the inert ointment/novel patch and oral pill/tablet in the placebo group than in the control group).	NA	NA	NA	Some concerns

^a^ In Liu's study, three categories (i.e., animal, tool and fruit) paired with low, high and medium pain intensities were counterbalanced across participants for CS_low_, CS_high_ and CS_control_. The table shows an example of CS_low_ and CS_high_ as well as CS_control_.

^b^ Zunhammer's study combined and analysed the data from six substudies, including the studies by Kessner and Hofmann, as well as other unpublished studies. Note that the findings were reported per group for the studies including both placebo and nocebo groups.

^c^ Studies were assessed as crossover studies in RoB2.

##### 3.2.1.1 Stimulus Generalisation of Placebo and Placebo‐Like Effects

###### 3.2.1.1.1 Behavioural Results

Four studies focused on generalisation of placebo effects at treatment level. Specifically, these four studies induced placebo hypoalgesia of placebo treatment (a patch or a cream) through combining verbal suggestion and conditioning, and then tested generalisation of placebo effects to a novel placebo treatment provided by the same route of treatment administration (i.e., ointment or a different patch) or a different one (i.e., oral pill, tablet or cold‐pack therapy) (Choi et al. 2022; Hofmann et al. 2014; Kessner et al. 2014; Zunhammer et al. 2017). The results of these four studies on generalisation of placebo effects at the treatment level are relatively equivocal. Two studies showed similar (or insignificantly less) placebo hypoalgesia upon administering the novel placebo treatments similar to the initial placebo hypoalgesic effects (Kessner et al. 2014; Zunhammer et al. 2017), and two studies did not find generalisation of placebo pain relief from initial placebo treatment to novel placebo treatments (Choi et al. 2022; Hofmann et al. 2014). Among these four studies, Zunhammer and colleagues' results (2017) were based on data that merged the two studies included (Hofmann et al. 2014; Kessner et al. 2014) and unpublished studies with inconsistent findings. In addition, it should be noted that participants in all these four studies included were told that the original and novel placebo treatments could relieve pain (Choi et al. 2022; Hofmann et al. 2014; Kessner et al. 2014; Zunhammer et al. 2017), which perhaps induced placebo effects on generalisation stimuli rather than pure generalisation effects.

Seven studies tested generalisation at the perceptual and categorical levels, after a combination of verbal suggestion and conditioning or conditioning alone induced placebo and placebo‐like effects on pain successfully (Kampermann et al. 2021; Koban et al. 2018; Liu et al. 2019). Three of these seven studies illustrated that placebo hypoalgesia could generalise from an initial, conditioned stimulus to novel, generalisation stimuli that consisted of pictures that varied perceptually (Kampermann et al. 2021; Koban et al. 2018), and three studies also reported similar placebo hypoalgesia with generalisation stimuli that varied categorically (Koban et al. 2018; Liu et al. 2019). However, one study by Koban and colleagues only found generalisation effects at the perceptual level for participants who were successfully conditioned initially: no generalisation effects were found when all participants were taken into account, including those who had not shown the conditioning effects (Koban et al. 2018). The lack of robust generalisation effects in this study may be due to the combination of too few learning trials and too many generalisation trials: the other two studies in the same article included more learning trials and fewer generalisation trials and did show generalisation effects. Furthermore, five out of seven studies showed that generalisation stimuli that were more similar to the initial conditioned stimulus evoked stronger pain alleviation than generalisation stimuli that were less similar to that stimulus, at either a perceptual or a categorical level (Kampermann et al. 2021; Koban et al. 2018; Liu et al. 2019). For instance, the results showed that the magnitude of pain relief with a novel facial cue was increased with increasing similarity to the conditioned cue, and the greatest pain relief was reported with the conditioned facial cue (Kampermann et al. 2021).

###### 3.2.1.1.2 Neurobiological Results

Only two imaging studies explored brain activity associated with generalisation of placebo hypoalgesia; these did not yield consistent findings. One study showed that stronger generalisation of placebo effects was linked with increased engagement of the right dorsolateral prefrontal cortex and the anterior cingulate cortex (Kessner et al. 2014), while the other study reported deactivation of the right inferior parietal lobule in the novel placebo treatment, although no generalisation effects were found with novel treatments (Choi et al. 2022). The regions mentioned in these two studies have been linked to pain‐related perception and expectancy‐induced placebo hypoalgesia (Benedetti and Piedimonte 2019).

###### 3.2.1.1.3 Moderators and Mediators of the Effects

Five studies examined moderators and/or mediators for the generalisation of placebo and placebo‐like effects, but no clear patterns were observed. The factors tested in the studies were expectancy, initial placebo effects and individual characteristics related to anxiety, depression, fear of pain, optimism, intolerance of uncertainty and internal‐external control (Kampermann et al. 2021; Kessner et al. 2014; Liu et al. 2019; Zunhammer et al. 2017). Only one study reported that stronger generalisation of placebo effects was associated with lower levels of trait anxiety and depression (Kessner et al. 2014), and two studies indicated that initial conditioning effects seemed to be associated with generalisation effects (Liu et al. 2019). In addition, the extent of similarity between initial and generalisation stimuli may also be considered as a moderator in generalisation effects (Kampermann et al. 2021; Koban et al. 2018; Liu et al. 2019). Given the limited number of studies and multiple factors tested across these studies, some caution is required when interpreting the associations observed.

##### 3.2.1.2 Stimulus Generalisation of Nocebo and Nocebo‐Like Effects

###### 3.2.1.2.1 Behavioural Results

Five studies induced nocebo‐like effects on pain by repeatedly surreptitiously pairing increased pain stimulation with conditioned images, and then examined whether generalisation stimuli (consisting of novel images) could increase pain as the conditioned stimuli did (Devriese et al. 2000; Koban et al. 2018; Liu et al. 2019). Three of the five studies reported less or similar nocebo hyperalgesia with generalisation stimuli than with the initial images, either at the perceptual level (i.e., images of Gabor angles) or at the categorical level (i.e., the category of animal, tool or vehicle) (Koban et al. 2018; Liu et al. 2019). One study found generalisation effects only when participants who had initially successfully learned nocebo‐like effects were considered (Koban et al. 2018), while one study reported no generalisation of nocebo hyperalgesia (Liu et al. 2019). Aside from pain, only two studies investigated other symptoms (Devriese et al. 2000; Quinn et al. 2015). One study examined nocebo‐like effects on breathing symptoms at the categorical level and tested whether novel odours could induce similar breathing symptoms (Devriese et al. 2000). Participants first smelt odours (conditioned stimulus) that were mixed with CO_2_‐enriched air (which could induce breathlessness, unconditioned stimulus) to associate the odours with breathing symptoms, and were then exposed to novel odours that were mixed with normal air from the room. The results showed that participants only learned the conditioning effects when the conditioned stimulus was a foul‐smelling odour. The conditioning effects were found to generalise only to similarly valenced odours, as participants reported more breathing symptoms elicited by novel foul‐smelling odours than by fresh‐smelling ones (Devriese et al. 2000). Another study focused on the generalisation of nocebo effects on nausea at the treatment‐context level (Quinn et al. 2015). Nausea symptoms were induced by active Galvanic Vestibular Stimulation (through passing small electrical currents to the vestibular end organs). Participants were randomised to receive either inactive stimulation in a control group or active stimulations in experimental groups (context‐consistent group and context‐change group) in a conditioning phase. All participants then were tested with the same inactive stimulation. The control and context‐consistent groups were tested in the same room as in the conditioning phase, and the context‐change group was tested in a novel room. The results showed similar nausea symptoms in both rooms, indicating successful generalisation (Quinn et al. 2015).

###### 3.2.1.2.2 Neurobiological Results

We are not aware of neuroimaging studies investigating stimulus generalisation of nocebo and nocebo‐like effects on somatic symptoms.

###### 3.2.1.2.3 Moderators and Mediators of the Effects

Just two studies thus far have explored factors associated with generalisation of nocebo‐like effects, and their findings show that expectancies and negative affectivity may modulate the generalisation of nocebo‐like effects (Devriese et al. 2000; Koban et al. 2018). Specifically, one study found generalisation effects only with participants who expected nocebo hyperalgesia with generalisation stimuli (Koban et al. 2018); the other illustrated that only participants with high negative affectivity reported generalisation effects to novel odours (Devriese et al. 2000).

#### 3.2.2 Response Generalisation of Placebo and Nocebo Effects

Of 10 studies investigating generalisation of placebo and nocebo effects from one response, e.g., perceived somatic sensation, to another response (see Table 2), six examined response generalisation of placebo effects, two examined response generalisation of nocebo effects, and two assessed response generalisation of both placebo and nocebo effects. Among these 10 studies, four studies featured generalisation within sensory modalities (e.g., from one type of pain to another type of pain), and the other studies featured generalisation across modalities (e.g., from pain to fatigue). In detail, eight studies investigated generalisation of placebo and/or nocebo effects from one type of pain (i.e., induced by heat, electricity, CO_2_ laser stimulator or an iontophoretic pain generator) to other types of pain (induced by an algometer or a blood pressure cuff), itch (induced by cowhage spicules), fatigue (induced by a finger flexor device) or negative emotion (induced by images). Two studies examined generalisation of nocebo effects from one type of itch (i.e., induced by electricity or cowhage spicules) to another type of itch (induced by histamine or mechanical stimuli) and/or to touch (induced by mechanical stimuli). Effect sizes for each study included are presented in Table 4.

**TABLE 4:** Overview of the findings of the studies included testing response generalization of placebo and nocebo effects on prevalent physical symptoms.

Study	Findings of originally induced effects	Findings of generalization effects	Std difference in means	SE	95%CI	Overall risk of bias
^a^ Bartels et al., (2017)	Nocebo effects on electrical itch were observed in part 1. Reduced nocebo effects upon counterconditioning were observed in part 2.	Generalization of the reduced nocebo effects upon counterconditioning from electrical itch to histamine‐induced itch was observed (i.e., histamine‐evoked itch intensities were significantly lower in the placebo group than in the nocebo group, and were not significantly different between the placebo group and the control group).	‐1	0.236	[−1.516, −0.484]	Some concerns
Carlino et al., (2016) experiment 1	Placebo effects on electrical pain tolerance were observed.	Generalization of placebo effects from pain tolerance to motor endurance was observed (i.e., the mean number of motor repetitions was significantly higher in the placebo group than in the control group).	2.833	0.448	[1.956, 3.71]	Some concerns
Carlino et al., (2016) experiment 2	No placebo effects on electrical pain tolerance was observed.	No generalization of placebo effects from pain tolerance to motor endurance was observed (i.e., the mean number of motor repetitions did not differ between placebo group and control group).	0.809	0.329	[0.164, 1.453]	Some concerns
Voudouris et al., (1989)	Placebo and nocebo effects on iontophoretic pain were observed.	No generalization of placebo effects from iontophoretic pain to ischemic pain was observed, but generalization of nocebo effects was observed (participants reported a decrease in mean pain tolerance with the placebo cream compared to no cream in the nocebo group, and a non‐significant decrease with the placebo cream compared to no cream in the placebo group.)	NA	NA	NA	Some concerns*
^b^ Weng et al., (2022b)	Placebo and nocebo effects on heat pain were observed.	Generalization of placebo and nocebo effects from heat pain to pressure pain was observed (i.e., the mean pain intensities were significantly lower/higher with the device ON than with the device OFF, in the placebo and nocebo group, respectively). No generalization to cowhage‐evoked itch was observed (i.e., the mean itch intensities did not differ between the device ON and OFF in both groups).	Placebo (within pain modalities)			Some concerns*
			0.638	0.194	[0.258, 1.019]	
			Placebo (across pain modalities)			
			0.041	0.177	[−0.306, 0.387]	
			Nocebo (within pain modalities)			
			‐0.333	0.179	[−0.684, 0.017]	
			Nocebo (across pain modalities)			
			‐0.261	0.177	[−0.608, 0.086]	
^b^ Weng et al., (2022a)	Nocebo effects on cowhage‐evoked itch were observed.	Generalisation of nocebo effects from cowhage‐evoked itch to mechanical itch was observed (i.e., mean itch intensities were significantly higher after the nocebo solution than after the control solution). No generalisation from cowhage‐evoked itch to mechanical touch was observed (i.e., the mean itch intensities did not differ between the two solutions).	Nocebo (within itch modalities)			Some concerns*
			‐0.24	0.153	[−0.54, 0.06]	
			Nocebo (across itch modalities)			
			‐0.239	0.153	[−0.539, 0.06]	
^c^ Zhang and Luo, (2009) experiment 1	Placebo effects on laser pain were not tested.	Generalization of placebo effects from laser pain to negative emotion was observed (i.e., the unpleasantness ratings were significantly higher with ON than with OFF).	0.455	0.263	[−0.06, 0.97]	High*
Zhang and Luo, (2009) experiment 2	Placebo effects on laser pain were not tested.	Generalization of placebo effects from laser pain to negative emotion was observed in the combination of verbal suggestion and conditioning (i.e., the unpleasantness ratings were significantly lower with ON than with OFF). EEG results are discussed on the section 3.1.2.	0.401	0.232	[−0.054, 0.857]	High*
Zhang and Luo, (2009) experiment 3	Placebo effects on laser pain were observed.	Generalization of placebo effects from laser pain to negative emotion was observed in the combination of verbal suggestion and conditioning (i.e., the unpleasantness ratings were significantly lower with ON than with OFF). EEG results are discussed on the section 3.1.2.	0.223	0.207	[−0.182, 0.628]	High*
^d^ Zhang et al., (2011)	Placebo effects on laser pain were not tested.	Generalization of placebo effects from laser pain to negative emotion was observed (i.e., the unpleasantness ratings were significantly higher with ON than with OFF). Note that fMRI results are discussed on the section 3.1.2.	2	0.333	[1.347, 2.653]	Some concerns*

^a^ Note that the design consisted of part 1 and part 2. Bartels investigated the counterconditioning of nocebo effects within itch modalities.

^b^ Note that the studies investigated response generalisation both within modality and across modalities, and the results of response generalisation within and across modalities were presented separately.

^c^ Note that in Zhang's paper (2009), one group in experiment 1 was not shown because no conditioning or verbal suggestions were given on initial laser pain, and only verbal suggestions were used on the generalisation response ‘emotion’; this does not meet our inclusion criteria.

^d^ Note that we deduced the results of the generalisation effects in a behavioural experiment from figures, as the behavioural experiment was only used for selecting the placebo responders (who learned placebo effects) to join the subsequent fMRI study and did not report initial placebo effects and generalisation effects. The findings of the generalisation effects in the fMRI study were not reported because the sample was limited to placebo responders (who learned placebo effects). Note that the findings were reported per group if the studies included both placebo and nocebo groups. A positive effect size indicates that a placebo effect was observe whiled, a negative effect size indicates that a nocebo effect was observed. The overall risk of bias was judged using the Cochrane Risk of Bias tool (RoB2). When both placebo and nocebo effects were compared, as well as when generalisation was assessed both within and across modalities, the RoB judgements were the same.

^e^ Studies were assessed as crossover studies in RoB.

##### 3.2.2.1 Response Generalisation of Placebo Effects

###### 3.2.2.1.1 Behavioural Results

Eight studies examined generalisation of placebo effects to novel responses within a modality or across modalities (Carlino et al. 2016; Voudouris et al. 1989; Weng et al. 2022b; Zhang et al. 2011; Zhang and Luo 2009). Two studies induced placebo hypoalgesia for one type of pain (i.e., heat pain, iontophoretic pain) with a placebo treatment via the combination of verbal suggestion and conditioning, and tested whether the same placebo treatment could also alleviate other types of pain (i.e., pressure pain, ischemic pain) without conditioning or verbal suggestions being applied directly to these novel pain stimuli (Voudouris et al. 1989; Weng et al. 2022b). Participants in one study reported lower pressure pain intensity (generalisation response) with the same placebo treatment than with the control treatment when tested immediately (Weng et al. 2022b), while another study reported non‐significant findings of ischemic pain tolerance (generalisation response) between the placebo trial and control trial when participants were tested on consecutive days (Voudouris et al. 1989). Six studies examined whether the learned placebo effects on pain could generalise to other symptoms: fatigue, itch or negative emotion (Carlino et al. 2016; Weng et al. 2022b; Zhang et al. 2011; Zhang and Luo 2009). Four out of six studies showed that placebo hypoalgesia could decrease other symptoms, regardless of whether these symptoms were tested for immediately or on consecutive days (Carlino et al. 2016; Zhang et al. 2011; Zhang and Luo 2009). One study that reduced pain by verbal suggestion alone did not lead to a generalisation response to fatigue (Carlino et al. 2016). It is worth noticing that in these five studies, verbal suggestion was applied not only to the initial pain but also to generalisation responses. Only in one of the six, participants did not receive learning methods directly related to the generalisation response and also did not find a generalisation response, i.e., from heat pain to cowhage‐evoked itch (Note that another generalisation response (i.e., pressure pain) was tested after the induction of placebo effects, but before cowhage‐evoked itch was tested) (Weng et al. 2022b).

###### 3.2.2.1.2 Neurobiological Results

Two imaging studies investigated the underlying neuroimaging mechanisms of the generalisation of placebo effects, from laser pain to negative emotion evoked by unpleasant pictures (Zhang et al. 2011; Zhang and Luo 2009). The electroencephalogram study showed that generalisation of placebo effects may be linked with increased N2 amplitude and decreased P2 amplitude, which implied that the unpleasant pictures likely induced the placebo expectancies that may modulate negative emotion via cognitive processing (Zhang and Luo 2009). The fMRI study reported an association between generalisation response (i.e., negative emotion) and reduced activity of the right amygdala, the right thalamus and the parahippocampal gyrus, which are also involved in regulating emotional processes (Zhang et al. 2011).

###### 3.2.2.1.3 Moderators and Mediators of the Effects

One study explored the role of affect and cognition (i.e., anxiety, stress, attention and catastrophizing) in response generalisation of placebo effects within the pain modality and from pain to itch but found no associations for any of them (Weng et al. 2022c).

##### 3.2.2.2 Response Generalisation of Nocebo Effects

###### 3.2.2.2.1 Behavioural Results

Two studies induced nocebo effects via verbal suggestion and conditioning on pain (i.e., heat pain and iontophoretic pain) and tested whether these nocebo effects generalised to other types of pain (i.e., pressure pain and ischemic pain) (Voudouris et al. 1989; Weng et al. 2022b). After the induction of nocebo effects on heat pain, participants reported higher pain intensities evoked by pressure pain (Weng et al. 2022b). Similarly, after the induction of nocebo effects on iontophoretic pain, participants reported decreased pain tolerance evoked by ischemic pain (Voudouris et al. 1989). In line with the observation of generalisation of nocebo effects within the pain modality, one study reported nocebo effects upon only verbal suggestions of increased cowhage‐evoked itch when applying a nocebo treatment, which generalised to mechanical itch when applying the same nocebo treatment, without direct verbal suggestions regarding mechanical itch (Weng et al. 2022a). Another study first induced negative expectations regarding the itch‐increasing effects of a sham electrode on electrocutaneous itch via verbal suggestion and conditioning (Bartels et al. 2017). Afterward, participants were allocated into three groups to investigate the reduction of these effects: (1) positive manipulation to counteract the previously learned nocebo effects, (2) negative manipulation to continue previously learned nocebo effects, (3) extinction group to extinguish nocebo effects (i.e., no information was given and all stimuli tested were applied at medium intensity). Effects were not only tested on electrocutaneous itch (initial response), but also on histamine‐evoked itch (generalisation response). Itch evoked by electricity and histamine was both significantly lower upon a positive expectation procedure than a negative expectation and an extinction procedure, indicating successful counterconditioning of nocebo effects on the initial sensation that generalised to the novel sensation (Bartels et al. 2017). In contrast, two studies did not find response generalisation of nocebo effects across modalities (Weng et al. 2022a, 2022b). One found nocebo effects induced via verbal suggestions on cowhage‐evoked itch, but effects did not generalise to mechanical touch (Weng et al. 2022a). Another study failed to find that nocebo effects that were induced by the combination of verbal suggestions and conditioning generalised from heat pain to cowhage‐evoked itch (Weng et al. 2022b).

###### 3.2.2.2.2 Neurobiological Results

We are not aware of neuroimaging studies investigating the response generalisation of nocebo effects.

###### 3.2.2.2.3 Moderators and Mediators of the Effects

Two studies explored the associations between expected and experienced generalisation responses, but reported non‐significant findings (Weng et al. 2022a, 2022b). Also, anxiety, stress, attention and catastrophising were not found to predict generalisation of nocebo effects within pain and itch modalities, nor across modalities (Weng et al. 2022c).

## 4 Discussion

This review provides an overview of the literature on stimulus and response generalisation of placebo and nocebo effects on prevalent somatic symptoms including pain, itch, dyspnea, nausea and fatigue. The 23 studies reviewed appear to consistently show that placebo and nocebo effects can generalise across stimuli and responses. Evidence further suggests that these generalisation effects are stronger when generalisation stimuli/responses more closely resemble the initial one. Across modalities, response generalisation remains unclear without repeated verbal suggestions. No clear conclusion can be drawn from the few neuroimaging experiments that investigated neurobiological mechanisms. Similarly, no consistent associations have been observed in the few studies exploring predictors or mediators of these effects.

### 4.1 Summary and Discussion of the Current Findings

Only a few studies have started touching on somatic symptoms other than pain (18 out of 23 studies), such as itch (2 studies), fatigue (1 study), nausea (1 study) or breathing symptoms (1 study). It would be fruitful to conduct more research on response generalisation for these somatic symptoms, especially for symptoms co‐occurring in common diseases. Generalisation stimuli investigated are slightly broader, including treatment‐related (e.g., placebos) and treatment‐unrelated stimuli (e.g., Gabor patches). Investigating stimulus generalisation related to treatments would be particularly valuable, given their ecological validity.

Regarding learning methods, the combination of conditioning and verbal suggestion was frequently and effectively employed in both stimulus and response generalisation research (14 studies). Conditioning alone was used only in stimulus generalisation research (7 studies). Only two studies used verbal suggestion alone, related to response generalisation (e.g., Carlino et al. 2016). More research is required to test the effectiveness of conditioning or verbal suggestion alone on stimulus and response generalisation. It remains to be seen whether other learning methods, e.g., observational learning, can establish generalisation.

Finally, although it seems obvious, initially placebo/nocebo effects are the premise for generalisation effects. Six studies did not report the results of initial placebo/nocebo effects, complicating the interpretation of results concerning generalisation effects. It may be that initial placebo/nocebo effects were absent, or that any such effects were too weak to generalise to novel stimuli/responses. Future studies are recommended to address this.

### 4.2 Exploring Potential Mechanisms

Research is only starting to reveal the underlying mechanisms. First, evidence suggests that similarity may drive generalisation of placebo/nocebo effects, with stronger effects when novel stimuli/responses are more similar to the original stimulus/response (e.g., Liu et al. 2019), aligning with findings of fear generalisation research (Dunsmoor and Murphy 2015) and elemental theories (for an extensive review see Wagner 2008). Wagner (2008) proposed that stimuli and responses are represented by various elements. Learning for each element of the conditioned stimulus or responses depends not only on the associative weight of an element alone, but also on the total associative weight of all elements. Investigating how individuals perceive and differentiate (dis)similarity of novel stimuli/responses to the initial one and how these become associated would be valuable for generalisation research. Relatedly, emotions such as anxiety may influence this process of generalisation, supported by findings in fear generalisation research that individuals with higher anxiety levels have a broader generalisation gradient (Dunsmoor and Paz 2015). Further research could consider, for example, inducing different levels of anxiety to understand the impact of anxiety on generalisation of placebo/nocebo effects.

Second, evidence hints at the role of expectancies in generalisation effects. For instance, novel placebo treatments for pain relief activate brain regions related to expectancy and pain‐related perception, which may also be active during initial placebo treatment (Hofmann et al. 2014). This is in line with the response expectancy theory ‐ (Kirsch 2018) and predictive coding (Büchel et al. 2014), which suggests that expectancies help predict their responses to future stimuli. Notably, the associations between self‐report expectancy and generalisation effects are rarely tested and results are inconsistent: i.e., one study reported modulation effects of expectancy on generalisation effects (Koban et al. 2018), but others showed no association (Weng et al. 2022a, 2022b). Despite this, expectancies could be generated in accordance with prior experiences and may drive similar placebo/nocebo effects on comparable stimuli/responses. More research into understanding the function of expectancy in generalisation effects is required.

Finally, memory may need to be mentioned, as novel stimuli/situations can be integrated into existing memories, requiring retrieval of relevant information (Xu and Südhof 2013). Research shows that sleep can optimise the active re‐processing of new information into existing memory networks, which may enrich the extraction of features and generalisation effects over time (Diekelmann and Born 2010). It is worthwhile to explore the impact of memory and sleep by using multiple test trials and/or test trials across different days.

### 4.3 Limitations

Our findings are limited by the low number of studies available on each type of generalisation of placebo/nocebo effects, especially concerning generalisation of nocebo effects. More studies are warranted to draw solid conclusions. The studies included also show heterogeneity in terms of somatic sensations studied, generalisation designs and stimuli that are used to induce placebo and/or nocebo effects. Therefore, current data were considered unsuitable to meta‐analyze. Additionally, although the risk of bias was judged to be low for most studies and in most domains of the Cochrane Risk of Bias tool (version 2), there were some concerns regarding the selection of reported results for all but one of the included studies and regarding the randomisation process in over half of the studies. Moreover, an overall high risk of bias was judged in 3 studies. Furthermore, circa half of the studies had a methodological limitation in that a verbal suggestion was applied to generalisation responses (12 out of 23 studies). This may have biassed the results, as it may have induced placebo/nocebo effects rather than generalisation effects on generalisation stimuli/responses. Another limitation is a potential publication bias, as null findings are less likely to be published and the current review only focused on published studies.

### 4.4 Future Research and Clinical Implications

Several additional directions for future research may be considered. First, more extensive learning likely enhances placebo/nocebo effects and thus facilitates their generalisation, supporting previous findings that learning shapes placebo hypoalgesia (Colloca et al. 2008). Future studies can investigate to what extent the quantity and quality of learning and generalisation trials contribute to the occurrence of generalisation effects. Second, an important next step is to examine strategies for amplifying generalisation of placebo effects, such as using appropriate reinforcement of the learned effect, and for attenuating generalisation of nocebo effects, for instance, through latent inhibition (preexposure to a to‐be‐conditioned stimulus) (Quinn et al. 2017). Also, temporal aspects of the initial and generalisation responses could be a point of focus; addressing whether generalisation occurs only after the learned response or also during the learning phase. Third, reproducible and rigorous neuroimaging studies should investigate the neurobiological mechanisms, such as by modulating brain activity associated with generalisation of nocebo effects. Furthermore, to reduce the risk of bias and facilitate future meta‐analyses, studies should adopt and clearly report methodological details, including blinding procedures, allocation concealment and pre‐specified analysis plans. Finally, future studies should explore possible predictors for generalisation effects, including interactions between generalisation effects and biopsychosocial and treatment factors.

Importantly, translational and clinical research needs to be considered. First, all the studies reviewed induced placebo and/or nocebo effects in a laboratory setting. It is not comparable to patients undergoing actual treatment within a lifetime history of treatments. To our knowledge, generalisation of placebo/nocebo effects has not yet been investigated in clinical samples. Investigating whether present findings also apply to clinical samples may help further understand generalisation in the clinic, including how treatment outcomes can be improved in patient samples with multiple comorbidities and how the impact of prior treatment history on subsequent treatment outcomes may vary, also across symptoms. This may help the clinical decision‐making process. Second, future studies may provide insight into minimising stimulus/response generalisation of nocebo effects by emphasising the differences between original and subsequent treatments/symptoms after prior treatment failure, respectively. For example, introducing a novel treatment scheme after treatment failure and verbally highlighting the dissimilarity may help optimise the efficacy of novel treatments for the same symptom. Finally, treatment‐contextual factors (e.g., healthcare settings) have also been found to directly influence the quality of therapeutic outcomes (Benedetti 2008). Future studies can investigate stressing similarity and distinction in contextual factors in anticipation of generalisation of placebo and nocebo effects, respectively, such as changing healthcare interior settings (e.g., in virtual reality) after treatment failure (Quinn et al. 2015).

## 5 Conclusions

The current review indicates that placebo and nocebo effects on prevalent somatic symptoms can generalise over stimuli and responses. This implies that placebo and nocebo effects can generalise across symptoms and treatments in clinical practice, which can help understand patients, especially among those who are being treated for varying conditions and/or undergo several treatment regimens. Future studies could make use of the progress in research into placebo and nocebo effects as well as generalisation research in other fields, using a multimethod approach (e.g., psychophysical and neuroimaging techniques) in both healthy subjects and patients. This would benefit the understanding of the mechanisms beyond these generalisation effects, ultimately addressing carryover effects of treatment outcomes and paving the way for improved interventions in clinical practice.

## Author Contributions

All authors contributed to designing the study. L.W. screened the articles and extracted the information from the articles. K.J.P. and A.I.M.v.L. checked the extracted information. L.W. drafted the manuscript. L.W., K.J.P., A.I.M.v.L. and A.W.M.E. critically revised the manuscript. All authors edited and approved the final manuscript.

## Conflicts of Interest

The authors declare no conflicts of interest.

## Supporting information


Figures S1–S2.
