# Supplementary material for: Generalisation of Placebo and Nocebo Effects: Current Knowledge and Future Directions
Source: Eur J Pain. 2025 May 8;29(6):e70018. doi: 10.1002/ejp.70018 (PMC12059979; doi:10.1002/ejp.70018)
Supplement: Supplementary file 1 — Figures S1–S2. [file EJP-29-0-s001.doc]

**Pubmed**

**#1 "conditioning, classical"[Mesh] OR "conditioning, operant"[Mesh] OR conditioning[Title/Abstract] OR "verbal suggestion*"[Title/Abstract] OR learning [Title/Abstract] OR expecta*[Title/Abstract]**

**#2 generali*[Title/Abstract] OR transfer*[Title/Abstract] OR "carry-over"[Title/Abstract] OR "carry over"[Title/Abstract] OR "carryover"[Title/Abstract]**

**#3 pain [Mesh] OR analgesi* [Mesh] OR hyperalgesi*[Mesh] OR fatigue*[Mesh] OR mental fatigue[Mesh] OR muscle fatigue[Mesh] OR "pruritus*"[Mesh] OR "antipruritic*"[Mesh] OR "nausea*"[Mesh] OR "motion sick*"[Mesh] OR "emetic*"[Mesh] OR antiemetic[Mesh] OR "dizziness"[Mesh] OR "vertigo"[Mesh] OR "syncope"[Mesh] OR "dyspnea*"[Mesh] OR "asthma*"[Mesh] OR pain[Title/Abstract] OR analgesi*[ Title/Abstract] OR hypoalgesia [ Title/Abstract] OR hyperalgesi*[ Title/Abstract] OR fatigue*[Title/Abstract] OR mental fatigue[Title/Abstract] or muscle fatigue[Title/Abstract] OR "itch*"[Title/Abstract] OR "prurit*"[Title/Abstract] OR "antipruritic*"[Title/Abstract] OR "nause*"[Title/Abstract] OR "motion sick*"[Title/Abstract] OR "emetic*"[Title/Abstract] OR antiemetic[Title/Abstract] OR "dizziness*"[Title/Abstract] OR "vertigo"[Title/Abstract] OR "syncope"[Title/Abstract] OR "faint*"[Title/Abstract] OR "dyspnea*"[Title/Abstract] OR "asthma*"[Title/Abstract]**

**#4 human NOT animal**

**#1 AND #2 AND #3 AND #4**

**672 papers**

**Rerun search strategy with (or without) adding the term “hypoalgesia”, 797 papers were found (the number of studies keep same with and without adding the term “hypoalgesia”). With limiting the date from 09/15/2022 to 01/21/2024, 104 papers were found.**

**Web of science**

**#1 All =(conditioning OR "classical conditioning" OR "operant conditioning" OR "observe*" OR "verbal suggestion*" OR learning OR expecta*)**

**#2** **TI =(generali* OR transfer*OR "carry-over" OR "carry over" OR carryover) OR AB =(Generali* OR Transfer*OR "carry-over" OR "carry over" OR carryover)**

**#3 TI =(pain OR analgesi* OR hyperalgesia* OR hypoalgesia OR fatigue* OR "mental fatigue" OR "muscle fatigue" OR itch* OR prurit* OR antipruritic* OR nause* OR "motion sick*" OR emetic*OR antiemetic OR dizziness* OR vertigo OR syncope OR faint* OR dyspnea* OR asthma*) OR AB =(pain OR analgesi* OR hyperalgesia* OR fatigue* OR "mental fatigue" OR "muscle fatigue" OR itch* OR prurit* OR antipruritic* OR nause* OR "motion sick*" OR emetic*OR antiemetic OR dizziness* OR vertigo OR syncope OR faint* OR dyspnea* OR asthma*)**

**#4 All = (human NOT animal)**

**#1 AND #2 AND #3 AND #4**

**623 papers**

**Rerun search strategy with (or without) adding the term “hypoalgesia”, 985 papers were found. With limiting the date from 09/15/2022 to 01/21/2024, 86 papers were found.**

**Psycinfo**

**TX ( conditioning OR "classical conditioning" OR "operant conditioning" OR "observe*" OR "verbal suggestion*" OR "verbal instruction*" OR learning OR expecta*)**

**AND**

**( TI(generali* OR transfer* OR "carry-over" OR "carry over" OR carryover) OR AB(generali* OR transfer* OR "carry-over" OR "carry over" OR carryover) )**

**AND**

**(TI (pain OR analgesi* OR hyperalgesia* OR fatigue* OR "mental fatigue" OR "muscle fatigue" OR itch* OR prurit* OR antipruritic* OR nause* OR "motion sick*" OR emetic*OR antiemetic OR dizziness* OR vertigo OR syncope OR faint* OR dyspnea* OR asthma*) OR AB (pain OR analgesi* OR hyperalgesia* OR hypoalgesia OR fatigue* OR "mental fatigue" OR "muscle fatigue" OR itch* OR prurit* OR antipruritic* OR nause* OR "motion sick*" OR emetic*OR antiemetic OR dizziness* OR vertigo OR syncope OR faint* OR dyspnea* OR asthma*) )**

**AND**

**TX ( human NOT animal )**

**618 papers**

**Rerun search strategy with (or without) adding the term “hypoalgesia”, 1047 papers were found. With limiting the date from 2022 to 2024, 117 papers were found.**

**Supplementary Figure 1. Risk of bias summary stimulus generalization studies: review authors’ scores about each risk of bias item for each included stimulus generalization study. P&N indicates that each domain and the overall risk of bias score applied to both placebo and nocebo groups. NA (not applicable) indicates that these studies with a parallel design did not assess the domain "bias arising from period and carryover effects." A green circle with a "+" indicates a low risk of bias, a yellow circle with a "!" indicates some concerns risk of bias, and a red circle with a "–" indicates a high risk of bias.**

**Supplementary Figure 2. Risk of bias summary response generalization studies: review authors’ scores about each risk of bias item for each included response generalization study.** P&N indicates that each domain and the overall risk of bias score applied to both placebo and nocebo groups. NA (not applicable) indicates that these studies with a parallel design did not assess the domain "bias arising from period and carryover effects." ^a^ Note that ratings for each domain and the overall risk of bias rating were applied to response generalization within and across modalities. A green circle with a "+" indicates a low risk of bias, a yellow circle with a "!" indicates some concerns risk of bias, and a red circle with a "–" indicates a high risk of bias.
